# Contemporaneous mobile- and stagnant-lid tectonics on the Hadean Earth

**DOI:** 10.1038/s41586-025-10066-2

**Published:** 2026-02-04

**Authors:** John W. Valley, Tyler B. Blum, Kouki Kitajima, Kei Shimizu, Michael J. Spicuzza, Joseph P. Gonzalez, Noriko T. Kita, Ann M. Bauer, Stephan V. Sobolev, Charitra Jain, Aaron J. Cavosie, Alexander V. Sobolev

**Affiliations:** 1 Department of Geoscience, University of Wisconsin–Madison, Madison, WI USA; 2 GFZ Helmholtz Centre for Geosciences, Geodynamic Modelling Section, Potsdam, Germany; 3 University of Potsdam, Institute of Geosciences, Potsdam, Germany; 4 Space Science and Technology Centre, School of Earth and Planetary Sciences, Curtin University, Perth, Australia; 5 Univ. Grenoble Alpes, Univ. Savoie Mont Blanc, CNRS, IRD, Univ. Gustave Eiffel, ISTerre, Grenoble, France; 6 Present Address: Amentum at NASA Johnson Space Center, Houston, TX USA; 7 Present Address: Research and Collections, New York State Museum, Cultural Education Center, Albany, NY USA

**Keywords:** Geochemistry, Tectonics

## Abstract

The first billion years of Earth history witnessed the emergence of continental magmatism, oceans and life. Yet, the details of how continents formed remain unknown because of the absence of preserved rocks^1–8^. Two conflicting Hadean models predominate: early onset of subduction and plate tectonics^2–4^, compared with early stagnant-lid and plume processes with delayed (post-Hadean) plate tectonics^5–7^. Here we report trace-element ratios (including Nb–Sc–U–Yb) correlated with age and hafnium and oxygen isotope ratios for Hadean detrital zircons from the Jack Hills (JH), Western Australia, which record unprecedented insights into the timing and setting of early magmatism. More than 70% of Hadean JH detrital zircons have Sc/Yb > 0.1, and 47% have U/Nb > 20, fingerprints for continental-arc and subduction settings. The remainder are ocean-island-like with little evidence for ocean-ridge settings. Hadean JH zircons probably originated from distinct terranes with separate tectonic histories. Subduction-related magmatism in the Hadean, as documented by JH zircons, alternated with periods of magmatic quiescence. This contrasts with dominantly stagnant-lid-like signatures for most Barberton Hadean zircons. The diverse settings for Jack Hills and Barberton detrital zircons imply contemporaneous operation of different tectonic styles during the Hadean, as well as a broader diversity of early crustal origins than previously known.

## Abstract (web summary)

Geochemical data from zircons show that subduction-like processes were operating contemporaneously with stagnant-lid-like processes at different locations as early as 4.4 billion years ago on the Hadean Earth.

## Main

Interpretations of Hadean tectonics are largely based on geochemistry of detrital zircons dated to more than 4 billion years ago (Ga), because no rocks older than 4.03 billion years (Gyr) have been identified^1,2^. The geochemical evidence is sparse and enigmatic^1–8^.

Most Hadean zircons are igneous, and their Ti concentrations suggest formation at moderate to low magmatic temperatures in the crust^2^. Mildly elevated oxygen isotope ratios record the presence of oceans and habitable conditions in the Hadean^9^. It is debated whether Hadean crust formed in subduction-like settings or was an immobile ‘stagnant lid’ of mafic to ultra-mafic composition; a spectrum of models has been proposed^2–8^. An important distinction is that during subduction, surface rocks such as hydrated basalt and serpentinite are drawn beneath the crust, promoting hydrous partial melting and production of granitoids^10^. This subduction does not require plate tectonics. In a stagnant-lid setting, ‘drips’ of dry granulite and eclogite sink from the base of the crust, with little water or granitoid production^8,11^.

Most Hadean zircons are detrital, separated from parent rocks by weathering and variably abraded by erosion, complicating tectonic interpretation. Some are fully rounded^1,12,13^ and possibly transported thousands of kilometres. Thus, apart from subduction versus stagnant lid settings, we investigate two other competing hypotheses: Hadean zircons originated from a single tectonic setting and possibly a small terrane, compared with Hadean zircons that originated from diverse tectonic settings through several events distributed over large continental areas comparable to known Archean terranes.

Constraining these questions is key for understanding the origin and growth of continental crust and the evolution of habitable surface environments suitable for the emergence of life. We test these ideas by evaluating key trace-element ratios (Nb, Sc, U, Yb), Hf and O isotopes, and age in the two largest known suites of Hadean zircons, the Jack Hills metasediments, Australia^1,2^, and the Green Sandstone Bed, Barberton Greenstone Belt, South Africa^13^ (BGSB).

## Subduction and genesis of granitoids

The characteristics of a subduction setting as identified by trace elements in zircon require clarification. Elevated ratios of U/Nb and Sc/Yb in zircon are indicative of magma-genesis associated with subduction in a continental-arc setting and hydrous melting^14^. However, subduction does not necessarily indicate modern-style plate tectonics, which includes relatively stable, long-lived subduction and continental drift of rigid plates. The conditions that define modern plate tectonics may have come much later than the earliest subduction. Other authors have referred to early subduction-like processes as semi-subduction, passive subcretion or local subduction^5,7^. The elevated trace-element ratios reflect intermediate composition granitoids, which are most likely to have been formed by hydrous partial melting of basaltic or komatiitic crust that was altered by aqueous processes on the surface of Earth and then subducted. Thus, subduction is fundamentally different from stagnant-lid processes such as sagduction^8,15^. The important distinction is that subduction buries rocks from near the surface of Earth that may be hydrated, whereas drips from the base of a thick stagnant-lid crust would be at granulite and eclogite facies and thus were anhydrous and unlikely to form substantial volumes of granitic melt^8,16^. Nor were they likely to have created substantial geochemical reservoirs that contribute characteristics of surface processes (that is, mildly elevated oxygen isotope ratios) to later melting events.

Granitoids also form by crustal reworking driven by plume heating and could result from deep burial or impact melting^4,17–21^, although their relative importance has been debated^1,2,19^. Trace-element discriminants do not distinguish among these processes of crustal recycling that produce continental-type zircons.

## Hf isotopes in zircon

**Fig. 1:**
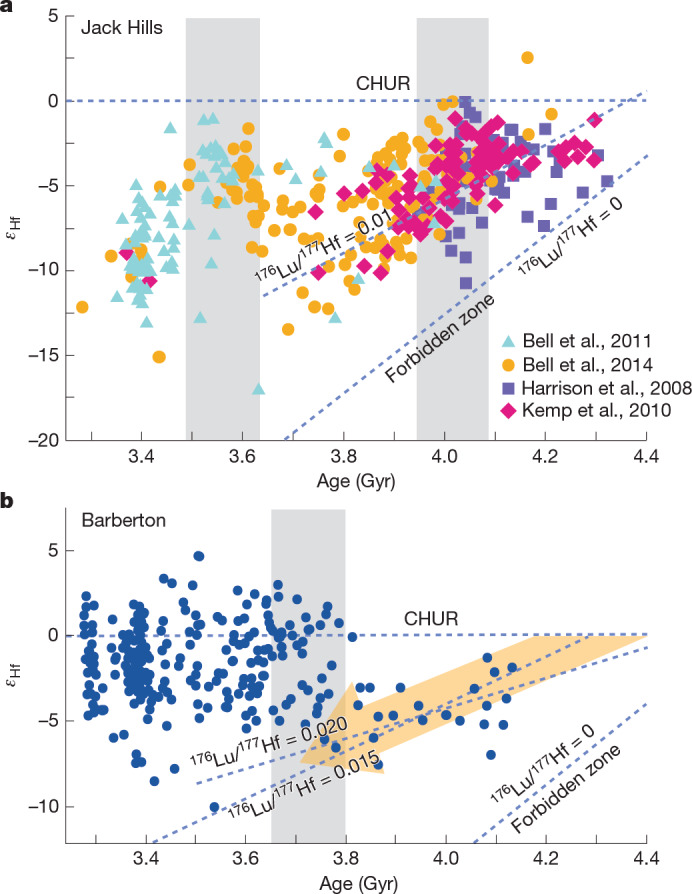
*ε*_Hf_ compared with age. **a**, Detrital zircons from JH^2^. Data from refs. ^11,24,46,47^. **b**, Detrital zircons from BGSB^13^. Starting in the Hadean, zircons from both regions fall on coherent trends corresponding to the reworking of Hadean protocrust with ^176^Lu/^177^Hf = 0.01–0.02. In BGSB, unradiogenic *ε*_Hf_ values persisted until about 3.8 Ga, indicating intra-crust reworking. Vertical trends and near-chondritic *ε*_Hf_ values (CHUR) after 3.8 Ga show input of juvenile material (grey band), consistent with the onset of subduction. The zircon *ε*_Hf_ trajectories from the JH are similar, but with two periods of juvenile input indicating subduction at about 4.0 Ga and 3.6 Ga.

The Hf-isotope ratios of zircons provide a robust record of primary magmatic differentiation (mantle extraction) that forms new crust and of melting within the crust (reworking) to form second-generation magmas. Many studies have reported Hf isotopes in zircons from Archean terranes (from less than 4 Ga)^11,22,23^. However, only two localities have yielded sufficient Hadean zircons to apply Hf isotopes along with age, trace elements and oxygen isotopes for reconstructing processes back to 4.3 Ga: JH^1,2,11,22–25^ and BGSB^13^ (Fig. 1).

A review of zircon Hf isotope data from six Archean cratons proposed that each of these terranes records a global change from stagnant lid to subduction at about 3.8–3.6 Ga (ref. ^23^). The oldest Archean zircons have negative *ε*_Hf_ values that tend to fall along radiogenic decay trends that project to mantle extraction of protocrust in the earliest Hadean followed by long periods of crustal residence and reworking. Chondritic values of *ε*_Hf_ near 0 (that is, CHUR) are indicative of primitive mantle magmas, but these are not found in Hadean zircons from BGSB (Fig. 1b) or in most studies of the earliest Archean (before 3.8 Ga). This shows that there was limited input of additional mantle-derived magma during crustal reworking in the Hadean. Some addition of primitive melts is permitted within the scatter of *ε*_Hf_, which may reflect magma mixing in the crust, but the amounts are limited by the absence of *ε*_Hf_ values near CHUR. The transition from these apparently stagnant-lid tectonics to subduction is proposed to have occurred globally in the interval 3.8–3.6 Ga, when all terranes show a resurgence of mantle-derived magmas with vertical mixing trends and chondritic *ε*_Hf_ values near 0 (refs. ^22,23^). The combined data for Hadean and Archean detrital zircons from BGSB^13^ (Fig. 1b) and JH^2^ (Fig. 1a) show step changes in *ε*_Hf_ that support this model. However, the JH suite is unique in that *ε*_Hf_ values near 0 are seen at both 4.0 Ga and 3.6 Ga, which are interpreted as separate crust-forming events^11,24^. Furthermore, the Hf isotope trajectories for JH zircons are relatively unradiogenic, and there are no positive *ε*_Hf_ values (as seen at <3.8 Ga in BGSB), suggesting that the mantle beneath the source terrane(s) of JH zircons was not as depleted as for BGSB.

## Trace elements in zircon

The trace element compositions of unaltered igneous zircons^12,26,27^ vary systematically among different tectonic settings, forming the basis of trace element signatures for tectonic settings. Discriminants based on element ratios reduce confounding effects, such as variable temperature, which strongly affect trace element distribution between zircons and magma^14,27–30^.

A previous study^14^ reported more than 5,400 secondary ion mass spectrometry (SIMS) analyses of igneous zircons of known parent rock to define the geochemical signatures of three broadly defined tectonic settings: mid-ocean ridge (MOR), continental-arc subduction (CAS, including magmatic arc and continental collision) and mantle plume-influenced oceanic islands (OI). These signatures are based on Phanerozoic zircons and are used hypothetically for Hadean zircons that may predate plate tectonics. There are no Hadean zircons in the dataset in ref. ^14^, because there are no known rocks older than 4.03 Gyr, and also no zircons from impact melts, which form a trace constituent of the crust. Nevertheless, ref. ^14^ demonstrates the strong discriminating power of Nb, Sc, U, Yb and other elements.

**Fig. 2:**
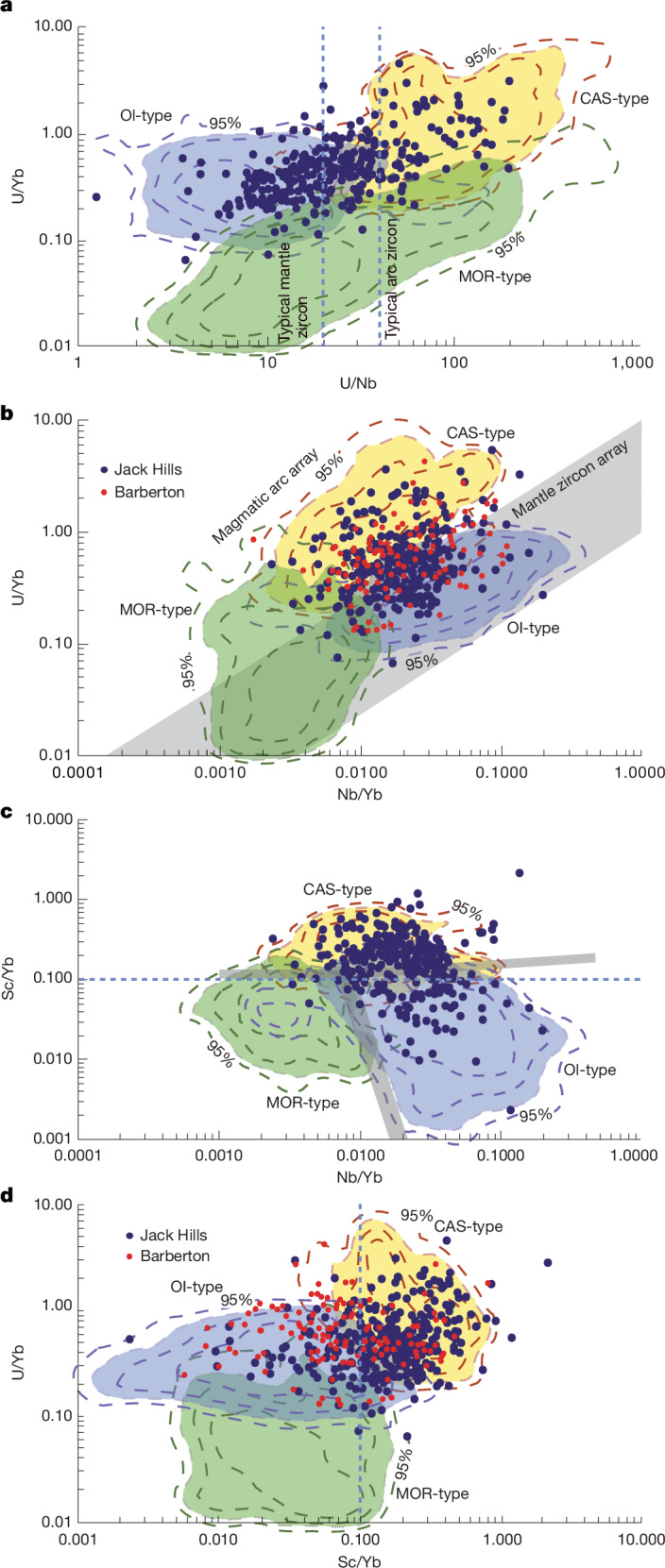
Zircon U, Nb, Sc and Yb discriminant diagrams. Zircon U, Nb, Sc and Yb discriminant diagrams (μg g^−1^/μg g^−1^) (ref. ^14^). The JH metasediments (this study, blue dots) and BGSB^13^ (red dots). Coloured fields and contours (50%, 80%, 90% and 95%) are for zircons from known magmatic source and tectonic setting (yellow indicates CAS-type; blue indicates OI-type; green indicates MOR-type^14^). **a**, U/Yb compared with U/Nb. **b**, U/Yb compared with Nb/Yb. **c**, Sc/Yb compared with Nb/Yb.** d**, U/Yb compared with Sc/Yb. Taken together, these diagrams show dominantly CAS- and OI-type compositions for both JH and BGSB zircons. Dashed blue lines show discriminants for CAS (higher ratios) versus OI settings.

The parameters controlling zircon composition are complex, including the composition of parent magma, coexisting minerals and thermodynamic conditions of formation^31,32^. For source rocks, the Nb/U_rock_ ratio does not change during dry melting in the mantle and thus is not affected by oceanic crust production^33^. The Nb/U_rock_ ratio is, however, fractionated by the hydrous partial melting of mafic compositions, which produces felsic melts in the presence of Nb-rich minerals such as rutile, leading to an enrichment of Nb relative to U in mantle cumulates compared with crustal magmas. In contrast to Nb-enriched mantle reservoirs, U is concentrated in the crust. The whole-rock values of Nb/U_rock_ range from about 6 ± 2 for continental crust to 47 ± 10 for OI and MOR basalts^33,34^. These canonical values have been incorporated in three-reservoir Earth models to estimate the extraction of low Nb/U_rock_ continental crust from primitive mantle and high Nb/U_rock_ oceanic basalts^33–37^. In zircon, ratios of U, Nb and Yb are expressed as U/Nb_zircon_ and U/Yb_zircon_ (inverted by convention^14^; ratios in this paper are for zircon unless followed by subscript ‘rock’), which is the ratio of a fluid-mobile element (U) and a relatively immobile element (Nb, Yb). These ratios are also tracers of aqueous alteration and recycling of crust. Thus, ratios of U/Nb_zircon_ and U/Yb_zircon_ are higher in CAS- than in OI- or MOR-type source rocks^14^ (Fig. 2a). However, crystallization of rutile (enriched in Nb) or monazite (enriched in U) may markedly affect the U/Nb ratio in coexisting zircon. Thus, the U/Nb ratio could be less distinctive for geodynamic settings than Sc/Yb, as described below.

Ratios of Sc/Yb in magmas are strongly affected by Sc fractionation into plagioclase and the ferromagnesian minerals amphibole and clinopyroxene. The crystallization of plagioclase in relatively dry OI basalts and MOR basalts, and amphibole in arc-related wet melts fractionate Sc/Yb differently. The partition coefficient of Sc between plagioclase and melt is two orders of magnitude higher than that for Yb^38^, whereas the partition coefficient of Sc between amphibole and melt is only a few times higher than that for Yb^39^. In zircon, Sc/Yb anti-correlates with SiO_2_ of the equilibrium melt, reflecting precipitation of coexisting minerals and the extent of crystallization^14,40^. Moreover, initially Zr-poor, arc-related CAS melts are likely to become zircon-saturated at higher SiO_2_ levels than OI basalts and MOR basalts, when their Sc/Yb ratios are considerably higher. Therefore, Sc/Yb ratios are consistently elevated in CAS-type zircons, which form in water-rich environments that facilitate hydrous melting and stabilize amphibole, relative to MOR- and OI-types^14^.

Step changes in Sc/Yb and U/Nb at 3.8 Ga were previously reported in Hadean and Eoarchean detrital zircons from BGSB, shifting from mantle-derived values (OI) in the Hadean to values typical of continental crust (CAS) in the late Eoarchean^13^. This appearance of arc-like (CAS) trace element signatures correlates with the step change of Hf isotopes at 3.8 Ga (Fig. 1b) and provides an independent record supporting the proposed global onset of subduction and termination of stagnant-lid tectonics^13,23^. Here, we report Sc/Yb and U/Nb ratios correlated with age and hafnium and oxygen isotope ratios from JH zircons to gain insight into Hadean and Archean tectonics. JH zircons span a longer timescale than those from BGSB, back to 4.4 Ga, and are uniquely suited for testing this model.

## Trace element discriminants for tectonic setting

**Fig. 3:**
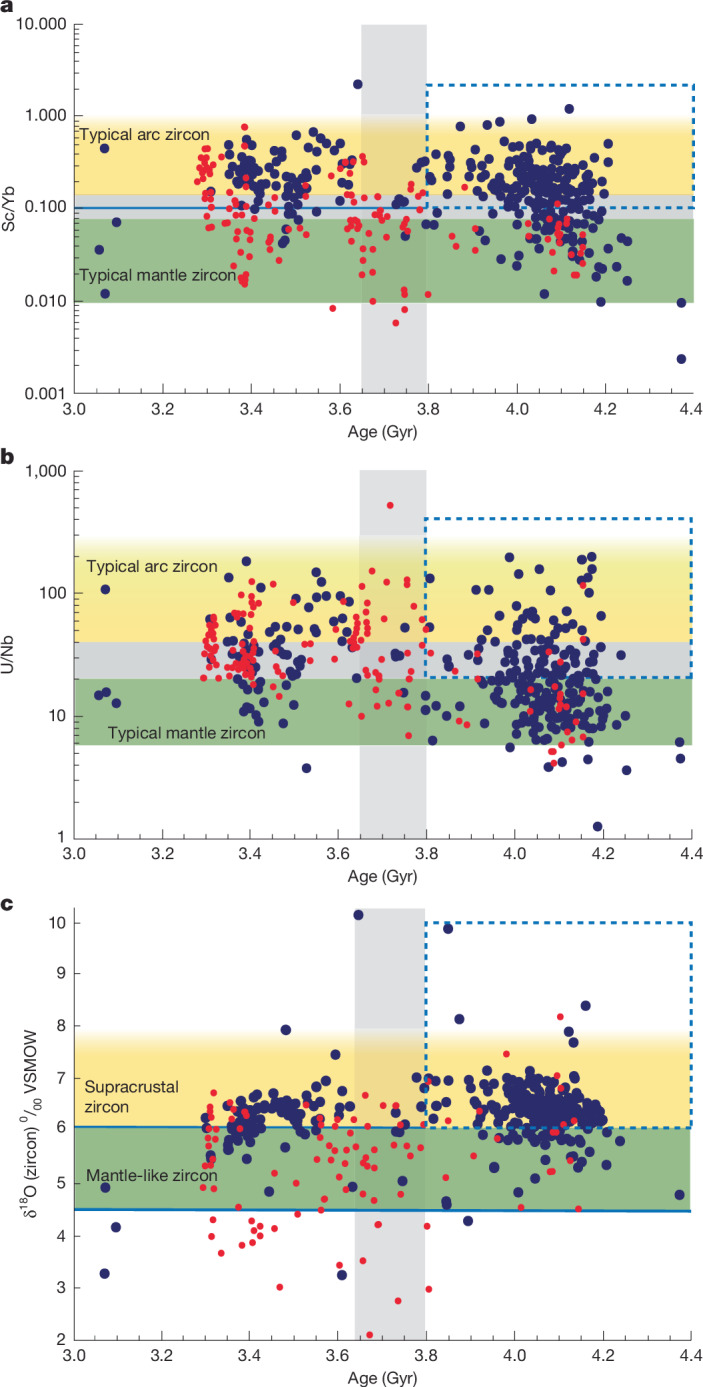
Sc/Yb, U/Nb and δ^18^O compared with age. Plots of Sc/Yb, U/Nb and δ^18^O compared with age for Hadean and early-Archean zircons from the JH metasediments (blue dots) (this study) and BGSB (red dots)^13^. The horizontal grey bands in **a** and **b** are regions of CAS–OI overlap. The vertical grey band is the time of change points seen for the BGSB data. The percentage of pre-3.8-Ga zircons that have CAS sources (inset, dashed outline; Table 1) is much greater for JH than for BGSB.

Trace element composition, age and oxygen isotope ratio were measured in selected domains of single zircons at the WiscSIMS Lab, University of Wisconsin–Madison, and are reported in Supplementary Table 1. Element ratios of Nb, Sc, Yb and U are plotted in Fig. 2a–d, with contoured fields for the three tectonic settings (MOR, OI and CAS)^14^. The continental (CAS) field is largely separated from the mantle (OI and MOR) at Sc/Yb = 0.1 (Fig. 2c,d). Furthermore, CAS zircons have U/Nb >20, and OI zircons have U/Nb <40 (ref. ^14^) (Fig. 2a,b). For high U/Yb, as in the JH and BGSB zircons, these ratios identify most CAS zircons with small areas of overlap among the fields, and for a relatively large zircon suite such as the JH, show distinct populations. Ratios of Sc/Yb > 0.1 (Fig. 2c,d) and U/Nb > 20 (Fig. 2a) are typical of zircons formed in continental-arc settings, although regions of overlap may contain OI-type zircons^13,14^ (Fig. 2). Figures 2a,c,d and 3a,b show zircons in these potentially ambiguous fields (shaded grey in Fig. 3a,b). Figure 2c shows cleaner separation and that most of the JH zircons from zones of overlap are CAS-type. These cut-offs are used here with recognition that up to 20% of the pre-3.8-Ga CAS zircons could be OIs.

The trace-element discriminant diagrams based on U/Nb and Sc/Yb (Fig. 2) show that the JH zircons are dominated by CAS- and OI-type compositions. It is notable that there are few MOR-type zircons. When Hadean and Archean samples are grouped together, JH and BGSB fall broadly within the same ranges (Fig. 2). However, the differences of Hadean zircons from the two terranes are apparent when Sc/Yb and U/Nb are plotted against age (Fig. 3). The JH Hadean suite is dominated by zircons with the CAS-type Sc/Yb-signature, whereas BGSB Hadean zircons are mostly OI-type. As shown in ref. ^13^, the BGSB suite records a distinct step change in proportions of the tectonic settings from mostly OI-type in the Hadean to about 50% CAS-type after 3.8 Ga (Fig. 3 and Table 1). The boxes in Fig. 3 outline CAS-like compositions for 4.4–3.8 Ga (Sc/Yb > 0.1, Fig. 3a; U/Nb > 20, Fig. 3b). In the JH suite, the proportion of >3.8 Ga zircons with Sc/Yb > 0.1 is four times greater (71%, *n* = 249) than in BGSB (18%) (Table 1). Likewise, 47% of the >3.8 Ga JH zircons have U/Nb > 20 compared with only 26% from BGSB. These new data show that the JH suite is distinctly different, with a high percentage of CAS-type Hadean zircons. This contrasts with BGSB, in which Hadean zircons with CAS-type compositions are far less common (Fig. 3a,b). Furthermore, at 3.8 Ga, the step changes from mantle-like to continent-like crust that are prominent in BGSB^13^ are smaller in the JH^41^. After 3.8 Ga, these ratios are more nearly the same in both zircon suites (JH compared with BGSB: 74% and 89% for U/Nb; 88% and 52% for Sc/Yb; Table 1). Thus, the JH detrital-zircon suite is, to our knowledge, the first and only known record showing a substantial component of continental-arc-like zircons in the Hadean.

**Table 1:** Comparison of JH and BGSB detrital zircons

	Sc/Yb > 0.1 (*n*)	U/Nb > 20 (*n*)	U/Nb > 40 (*n*)	δ^18^O > 6.1‰ (*n*)
JH metasediments				
>3.8 Ga	71% (254)	47% (238)	17% (238)	76% (242)
<3.8 Ga	88% (109)	74% (89)	36% (89)	64% (103)
BGSB				
>3.8 Ga	18% (17)	26% (19)	5% (19)	40% (20)
<3.8 Ga	52% (93)	89% (91)	40% (91)	22% (97)

Percentages of CAS-type zircons (Sc/Yb > 0.1, U/Nb > 20 or U/Nb > 40, and δ^18^O > 6.1‰) grouped by age from the JH (this study) and BGSB^13^. *n* indicates the number of zircons analysed.

Figure 3 and Table 1 show that the Sc/Yb proxy identifies a larger percentage of CAS zircons than U/Nb. Extended Data Fig. 3 shows this population of Hadean JH zircons with CAS-like Sc/Yb, but mantle-like U/Nb. This seeming Sc–Nb mismatch is most prominent in the Hadean and demonstrates that different processes fractionate these elements, and probably results from the presence of amphibole, which fractionates Sc, during hydrous melting.

## Oxygen isotope ratios

The δ^18^O(zircon) values are, on average, higher throughout the Hadean and Eoarchean in JH compared with BGSB; 76% of the pre-3.8-Ga JH zircons have δ^18^O > 6.1‰ compared with 40% of the pre-3.8-Ga BGSB zircons (Fig. 3c and Table 1). The higher δ^18^O values indicate hydrous alteration and reworking of near-surface material^9,42^. They are consistent with the more evolved CAS-type trace element signatures in the JH zircons and reflect the stronger local control of crustal contamination on magmatic δ^18^O than on trace elements and *ε*_Hf_ (ref. ^42^).

## Hadean and Eoarchean tectonics

The Sc/Yb and U/Nb signatures reported here provide, to our knowledge, the first direct evidence for different tectonic domains in the Hadean. Both JH and BGSB suites contain zircons from OI-type parent rocks before 3.8 Ga. But the JH suite is 71–47% CAS-type, unlike BGSB zircons, which are only 26–18% CAS-type. This diversity is unlikely to have come from one tectono-magmatic setting. The simplest interpretation is that the JH and BGSB sediments sampled two or more different Hadean terranes, each with a different and variable component of granitic crust. To maintain this bimodality during erosion would require either different source terranes on different continents or different terranes within a single large land-mass in the Hadean.

The change points at 3.8 Ga for trace elements in BGSB zircons (Fig. 3a,b, red dots) coupled with a vertical trend of increasing *ε*_Hf_ to near-CHUR values (Fig. 1b) were proposed to represent the end of stagnant-lid tectonics and the global onset of subduction with a mobile lid^13^. Similar vertical mixing trends in *ε*_Hf_ are common in new crust generated by Phanerozoic subduction^43^. Both JH and BGSB zircon-source terranes record sharp increases of *ε*_Hf_ at 3.6 Ga and 3.8 Ga, respectively, documenting the introduction of relatively juvenile material, within the time window proposed for the global onset of subduction^23^. However, there are important differences shown by trace element ratios that, in addition to Hf, demonstrate a more complex tectonic history. At about 4.0 Ga and again at 3.6 Ga, some JH zircons record relatively juvenile *ε*_Hf_ signatures and vertical mixing trends (Fig. 1a). Thus, the JH Hf data show mantle extraction of protocrust in the early Hadean (by CAS or OI processes), crustal reworking with little mantle input for 200–300 Myr, a period of more active subduction at 4 Ga followed by a second period of crustal reworking with little mantle input, and finally renewed subduction at 3.6 Ga. Whereas the BGSB zircons are consistent with a single protracted stagnant-lid-like period of crustal reworking that ended with the onset of subduction at 3.8 Ga, the JH zircons record two overlapping cycles dominated by reworking (about 4.4–3.6 Ga and 4.0–3.6 Ga) and two main periods of more-intense subduction (about 4.0 Ga and 3.6 Ga). Furthermore, the main inputs of juvenile mantle-derived magmas in the Eoarchean, as seen by zircon geochemistry (Figs. 1 and 3) are at different times, 200 Myr apart; 3.8 Ga for BGSB compared with 3.6 Ga for JH. Thus, the setting and timing of magmatism in the zircon-source terranes for JH and BGSB were distinctly different.

Geodynamic modelling provides constraints for tectonic interpretations of these results and has shown that more complex, polycyclic scenarios are realistic^4^. The results of our preferred high-mobility model^37,44^ show an oscillating (or fluctuating) mobile-lid regime characterized by periods of high crust mobility (driven by plume-induced subduction) that alternate with periods of low mobility squishy- or stagnant-lid-like regimes. In these models, most new mafic crust is associated with plumes rather than passive spreading centres. Subducting layers of hydrous near-surface rocks sink in punctuated events^37^ (Extended Data Fig. 2d, inset). Granitoids form from by melting of hydrated basalts in the lower part of the thick oceanic crust under amphibolite-facies metamorphic conditions. Similar to today, the primary water source is dehydration of subducted slabs. Crustal reworking could form granitoids with crustal geochemical characteristics mixed with OI-type precursors, such as those shown here to be common among JH zircons, without new input of juvenile uncontaminated mantle magmas. Importantly, these models show that both mobile-lid- and stagnant-lid-type regimes could have operated simultaneously in different terranes and are consistent with zircon ages and geochemistry (*ε*_Hf_, δ^18^O and trace elements) from both the JH and BGSB zircon-source terranes.

The multi-cycle history of reworking and subduction shown here for the JH zircon-source region(s) is not consistent with prevalent models of continuous stagnant-lid tectonics in the Hadean and a global transition to subduction in the Archean. A more nuanced and flexible model of Hadean tectonics is required to explain Hadean zircon data. As proposed here, both subduction-like and stagnant-lid-like processes operated simultaneously, but in different places. These results reconcile Hadean interpretations from Barberton komatiites^37^ that propose extensive subduction based on ^87^Sr/^86^Sr and Nb/U_rock_ in olivine-hosted melt inclusions, whereas mostly stagnant-lid-type BGSB zircons are reported^13^. These magmatic suites formed in different places; BGSB detrital zircons were transported from distant source terranes. Thus, the different interpretations of data from melt inclusions in olivine compared with zircon geochemistry further support a Hadean Earth with variable tectonic settings operating over large land masses. Recent comparison of U/Nb and Sc/Yb signatures in Archean zircons from known rocks of the Acasta Gneiss Complex and the Saglek-Hebron Complex, Canada, also shows contemporaneous operation of stagnant-lid and mobile-lid tectonics in the Eoarchean^45^, further supporting our tectonic interpretations. Subduction in the Hadean–Eoarchean was locally variable and short-lived, as predicted by the geodynamic model (Extended Data Fig. 2) and unlike the continuous and stable subduction characteristic of Phanerozoic plate tectonics. Furthermore, it is possible that less intense periods of active subduction occurred several times in the JH source terranes, producing the population of arc-like zircons from the late-Hadean. The change points seen at 3.8–3.6 Ga may mark large-scale stabilization of continental crust, rather than the onset of subduction. The Hadean eon as documented in JH zircons included multiple episodes of early mobile-lid tectonics, which represents a unique phase of Earth history that was more complex than previously thought.

## Methods

### Sample preparation, imaging and rejection criteria

The zircons in this study came from JH samples of Jack Hills metaconglomerate from the Discovery Outcrop on Eranondoo Hill^1^, from outcrops up to 900 m to the east, and from one cross-bedded metasandstone (01JH-36). Samples were reduced to sand size by electropulse disaggregation. Zircons were concentrated hydraulically on a gold table and by heavy liquids before separation of the low magnetic susceptibility zircon fraction by Frantz^48,49^. Zircons were hand-picked by binocular microscope, cast into 25.4 mm diameter epoxy mounts with analysis standards, and ground or polished at their approximate mid-sections.

The JH zircons of this study are divided into two suites. Sample mounts JH- and W- (01JH-13(a,b), 01JH-36, 01JH-42, 01JH-47, 01JH-54(a,b), 01JH-60(a,b) and W-74-3 and W-74-4) were extensively studied previously^1,12,26,50,51^ and in this study. Samples 01JH-12 and 03JH-141 were collected 1 m from 01JH-13, 01JH-54, W-74-3, and W-74-4. Zircons in mounts ERC-2 through ERC-10 were separated from 01JH-12 in 2022.

Before mounting, the zircons in mounts ERC-2 to ERC-10 were experimentally heated to 1,100 °C at 0.4 GPa of Ar atmosphere for 6 h in a rapid-quench internally heated gas apparatus to fuse melt inclusions for subsequent study. The zircons in sample mounts with no prefix ERC were not experimentally heated. Differences are observed in levels of retained radiation damage seen by Raman (effective dose) and cathodoluminescence, none of which influence the results reported here. No differences are observed between experimentally annealed zircons and not annealed zircons for age, oxygen isotope ratio or trace elements in the selected low-magnetic-susceptibility zircons with >95% concordant U–Pb ages^52^. This is consistent with many studies of chemically abraded zircons that were experimentally heated to 800–1,100 °C or above^53^ and further attests to the refractory and retentive nature of crystalline zircons.

Procedures for SIMS analysis of age (U–Pb), oxygen isotope ratio (δ^18^O and ^16^OH/^16^O) and trace element concentrations (REEs, Nb, Sc, Al, P, Ca, Ti, Fe, Y, Hf, Ta, Th and U) have been described elsewhere and are summarized below. A new aspect of these data is the analysis of Nb, Sc and Ta in zircon, which requires higher mass resolution than routinely used in studies with forward-geometry SIMS instruments^30^.

The polished mid-sections of zircons were imaged optically and by SEM (back-scattered electrons, secondary electrons and cathodoluminescence) before and after SIMS analysis. Pre-SIMS imaging allowed selection of analysis spots to avoid obvious alteration and inclusions, and targeting of sub-domains in zoned zircons. Post-SIMS imaging evaluated whether pits were irregular (that is, placed on inclusions, cracks and alteration zones) and in the selected domain^50,54,55^. Data from irregular pits were not considered further.

Zircon analyses were further filtered by composition. Acceptance criteria were conservatively set as follows: >95% concordant U–Pb ages; low concentrations of non-formula elements (<30 µg g^−1^ Al or Ca; <50 µg g^−1^ Fe); La < 0.25 µg g^−1^; Pr_*N*_ < 10; (Sm/La)_*N*_ > 10; Th/U > 0.1; Ce/Ce* > 3; and for δ^18^O, ^16^O/^1^H^16^O < 0.0004 (refs. ^26,50,55–59^). These zircons all passed the LREE-I test^55^, which is less stringent.

### Geochronology

All SIMS analyses were made by CAMECA IMS 1280 in the WiscSIMS Lab, University of Wisconsin–Madison. Zircons in mounts ERC-8, ERC-9 and ERC-10 were surveyed for quick ages in automatic mode. Samples were cleaned and carbon-coated to minimize common lead contamination and a Hyperion-II RF-source produced a 5-nA ^16^O_2_^−^ beam focused to around 15 μm on the sample surface. Simultaneous analysis of ^204^Pb^+^, ^206^Pb^+^, ^207^Pb^+^ and ^92^Zr_2_O^+^ took about 1.3 min per analysis (Supplementary Table 1). These quick model ages are based on ^207^Pb/^206^Pb.

Full U–Pb analyses were measured in this study for selected zircons in the ERC-2 to ERC-10 mounts. The data for JH and W mounts are compiled from earlier sources^1,12,26^. The ERC mounts were repolished to remove carbon and coated with Au for full U-Pb analysis. Gold coating (rather than C) yields higher and more stable count rates on Pb, but can include Pb from the Au, which must be removed by pre-sputtering before analysis and corrected for common Pb^60^. The primary beam of ^16^O_2_^−^ (Hyperion-II RF-source) was accelerated at 10 keV for an impact energy of 23 keV and a spot size of about 20 μm. An oxygen leak was used at a chamber pressure of about 1–2 × 10^−5 ^mbar to improve the Pb yield. Each counting cycle proceeded through the masses: ^92^Zr_2_^16^O^+^ [C], ^92^Zr_2_^16^O^+^ [L1], 200.5 (blank), simultaneous collection of Pb isotopes ^204^Pb^+^, ^206^Pb^+^ and ^207^Pb^+^, simultaneous collection of ^238^U^+^ and ^238^U^16^O^+^ and ^238^U^16^O_2_^+^ at MRP ~ 8,000. A total of six cycles were measured and the last five were integrated for age calculations. For all sessions except January 2024, the measured values of ^206^Pb/^238^U were corrected based on the ^206^Pb/^238^U compared with ^238^U^16^O_2_/^238^U trend as measured on reference zircon 91500 (refs. ^61,62^), which was also used to determine and correct the instrumental mass fractionation of Pb isotopes. In January 2024, calibration of U–Pb isotope ratios used zircon reference material M127 (ref. ^63^), which was also used to determine and correct the instrumental mass fractionation of Pb isotopes. Temora-1, Temora-2 (ref. ^64^), 91500 and M127 were run as secondary reference materials in each session. Measured ^204^Pb was used to correct for common Pb using the two-stage Pb evolution model in ref. ^60^. Ages are based on ^207^Pb/^206^Pb in the >95% concordant analyses.

### Oxygen isotope ratio and OH/O

Oxygen isotope ratios and ^16^O^1^H/^16^O (OH/O hereafter) were measured in three sessions for the ERC mounts. The data for 01JH and W mounts are compiled from earlier sources^1,26^. Analysis conditions were the same as described previously^26,65–67^. The primary beam of ^133^Cs^+^ was accelerated at 10 keV and focused to an approximately 10 μm diameter spot (about 1 μm deep) on the gold-coated sample surface with an impact energy of 20 keV. The ions of ^16^O^−^, ^16^O^1^H^−^ and ^18^O^−^ were analysed in multi-collector mode using three Faraday cup detectors. Primary beam currents were about 2 nA. Secondary ion yields for ^16^O^−^ varied from 1.43 Gcps nA^−1^ to 1.57 Gcps nA^−1^ and were nearly constant within each session. Analyses of zircon with ion yields more than 5% different from bracketing zircon standards are rare and were discarded. Four analyses on the UWZ1 zircon standard (δ^18^O = 4.96‰ Vienna Standard Mean Ocean Water (VSMOW); ref. ^68^) were made before and after every 10–15 sample analyses. The average precision for spot-to-spot analyses of δ^18^O on standards averages 0.15‰, 0.24‰ and 0.18‰ (2 standard deviations), respectively, for the three sessions. The measured ratios of ^18^O to ^16^O are corrected for instrumental bias as calculated from bracketing UWZ1 zircon analyses and reported in standard δ^18^O notation relative to VSMOW. No correction is necessary for matrix effects because of variable [Hf] in these zircons that mostly range from 7,000 μg g^−1^ to 12,000 μg g^−1^ (maximum = 15,723, average = 9,508 ± 1,244 μg g^−1^ for 410 analyses), which is close to UWZ1 (10,187 μg g^−1^) or KIM5 (8,960 µg g^−1^), but substantially higher than 91,500 (6,030 µg g^−1^; ref. ^68^).

High background-corrected values of OH/O can correlate to erroneous values of δ^18^O in altered zircon^26,59,69^. This correlation is also reported in ref. ^70^, which corrected OH/O with long-term averages in more heavily damaged JH zircons. Background correction in this study is based on the nominally anhydrous standard (UWZ1) analysed approximately every 2 h, and better describes improving vacuum conditions after each sample change. Values of OH/O > 0.0004 were rare in zircons that passed other acceptance tests and were rejected.

### Trace elements

Recent advances facilitate routine and accurate trace-element analysis of zircon by SIMS, including new protocols for high mass-resolution analysis that is required for Nb and Sc^30^; new zircon reference materials; smaller spot sizes (1–10 μm diameter); and tests to identify altered domains.

Trace and minor elements were measured in five sessions using magnet-scanning automatic-analysis mode by CAMECA IMS 1280 at WiscSIMS: Al, P, Ca, Sc, Ti, Fe, Y, Nb, La, Ce, Pr, Nd, Sm, Eu, Gd, Tb, Dy, Ho, Er, Tm, Yb, Lu, Hf, Ta, Th and U. An additional 12 masses were counted for purposes of calibration, to evaluate interferences and to keep the mass spectrometer tuned. A Hyperion-II RF-source provided the primary ^16^O^−^ beam that was accelerated at 10 keV with no energy offset for an impact energy of 23 keV and a beam current of about 5.0 nA. Spot sizes are about 15 μm in diameter. Secondary ions were detected with the axial ETP electron multiplier, except major elements (^28^Si, ^90^Zr), which were counted by the Faraday cup detector.

The mass spectrometer was tuned with an MRP ≈ 12,500, as measured on ^93^Nb. Quantification of peak shapes and direct measurement of ^92^ZrH^+^ allow for the estimation of backgrounds for limiting interferences (^93^Nb^+^ with ^92^ZrH^+^, and ^45^Sc^+^ with ^90^Zr^2+^; ref. ^30^). ^92^ZrH^+^ signals are dependent on several factors, including chamber pressure and the hydration state of the target zircon domain. For analyses in this study, ^92^ZrH^+^ contributions to ^93^Nb^+^ are a maximum of 0.17 µg g^−1^, and average 0.0079 µg g^−1^; relative signal levels average <0.2% of the peak value, with a maximum of 2%. ^90^Zr^2+^ is not measured directly, as Zr^2+^ is expected to be relatively consistent from analysis to analysis. ^90^Zr^2+^ backgrounds on ^45^Sc^+^ are calculated to be to be a maximum of <0.009 µg g^−1^, with an average 0.007 µg g^−1^, and have relative contributions averaging 0.04%, with a maximum of 1.1%.

At higher MRP, accurate mass calibration is especially sensitive to small changes in magnetic field and is aided by maintaining precise timings of magnet scans, and monitoring or adjustment of the mass calibration. In this study, tuning was maintained for several days with a new monitoring and correction routine that tracks and makes regular ppm-level adjustments to the magnetic field after every analysis. This routine monitors the position of five major-element peaks (^28^Si^+^, ^90^Zr^+^, ^90^Zr^16^O_2_^+^, ^90^Zr_2_^+^, and ^90^Zr_2_^16^O_3_^+^) with sensitive measurements on the peak and its flank and adjusts the magnet calibration to keep both reference and interpolated masses centered^30^.

Each analysis included a 30-s pre-sputter, centring of the secondary beam, and five cycles of counting from low to high mass. Count rates change more rapidly early in each analysis. Thus, the last four cycles are normalized to ^28^Si (on a cycle-by-cycle basis) and averaged for use in calculating concentrations.

Calculation of trace element concentrations is based on the conversion of normalized count rates between zircon and the glass standard NIST610 that was analysed regularly throughout each session. For most elements, this calculation incorporates a correction factor based on the comparison of trace element concentrations measured in zircon reference materials. Five reference materials were analysed: NIST 610 glass^71^, and four zircons, 91500 (refs. ^62,72^), MAD-559 (ref. ^72^), M127 and GZ7 (ref. ^73^) (Supplementary Table 2). Oxygen isotope zircon standards (UWZ1 and KIM5) were also analysed to test instrument conditions, but these zircons are not sufficiently homogeneous in trace elements to be used for calibration. We note that ref. ^14^ calibrated SIMS data with a combination of zircon reference materials CZ3 and MAD-1. We use MAD559, which was intercalibrated with these materials in ref. ^72^. In this study, we used the original discriminants^14^. This is consistent with published studies for BGSB^13^ and other zircons^13,41,45^. A previous study^40^ suggests revision of ref. ^14^ Sc/Yb and U/Nb discriminants. These differences are small and within analytical uncertainty.

Each analysis in our EarthChem dataset has calculated trace element concentrations in µg g^−1^, and for the lanthanides, the chondrite normalized values are also shown^74^. The measured compositions of U and Th are used in this study in agreement with the data in ref. ^14^ and published BGSB data^13^. If compositions of U were corrected for radioactive decay since the time of crystallization of Hadean zircon, they would be nearly twice as high, which would move U/Nb ratios away from the OI field if discriminant boundaries are not similarly adjusted, and making more JH zircons appear to plot in the field of continental crust.

### Age distribution in sample 01JH-12

**Extended Data Fig. 1:**
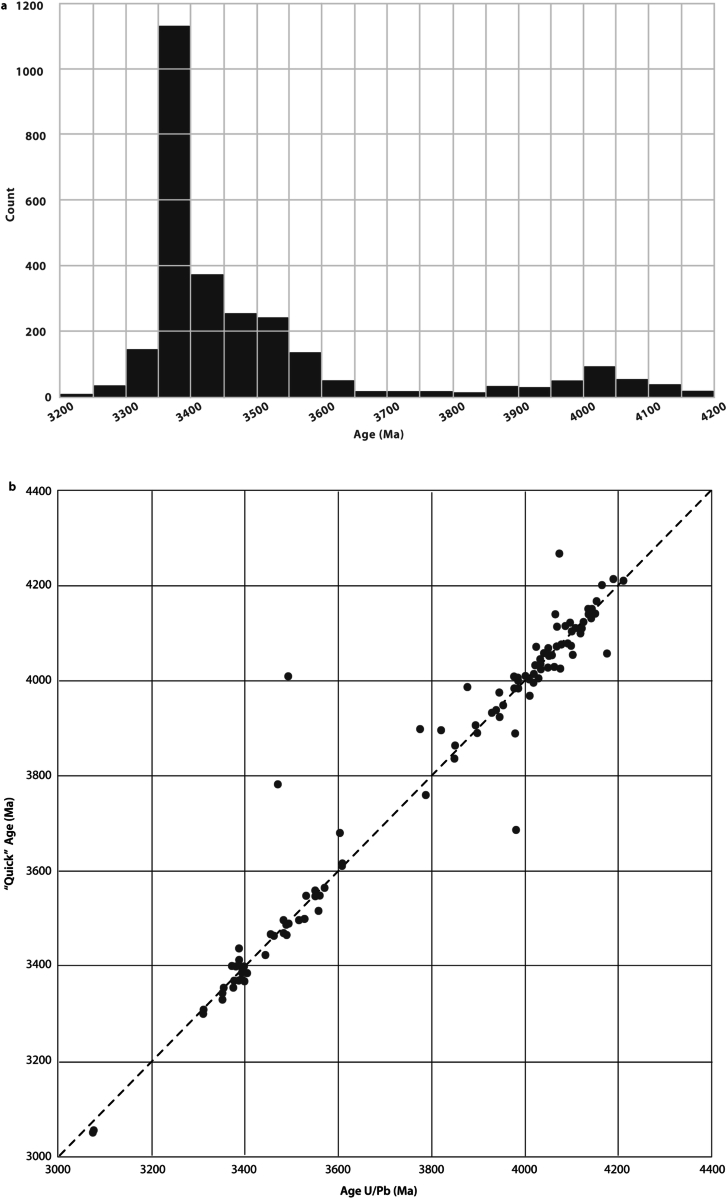
Geochronology of Jack Hills Zircons. Geochronology for Jack Hills detrital zircons from 3 of the 19 zircon mounts analyzed in this study. **1 A:** Histogram of ^207^Pb/^206^Pb ages from 1-min analyses, “quick ages”. **1B:** quick ages vs. ^207^Pb/^206^Pb ages from full U/Pb analyses. The 2839 quick ages represent all zircons in three epoxy mounts (ERC-8, -9, −10) showing the age distribution of JH zircons after sorting for low magnetic susceptibility by Frantz and hand-picking. The 125, >95% concordant U-Pb analyses, in addition to those reported earlier^12,26^, represent the most accurate ages for zircons analyzed in this study.

The percentage of Hadean zircons separated from different samples of JH metasediment in different studies varies greatly. It is important to recognize that no suite of detrital zircons is necessarily representative of their source rocks. Sorting occurs in nature because of the differences in erosion and possible selective destruction of metamict or otherwise damaged grains. Sorting also occurs in the lab because of heavy mineral concentration, magnetic sorting, hand picking, imaging and selection of analysis spots. To obtain the distribution of ages for selected zircons from epoxy mounts in this study, all zircons in 3 of the 19 mounts examined (ERC-8, ERC-9 and ERC-10; from sample 01JH-12 at the Discovery Outcrop) were dated by quick analyses (Extended Data Fig. 1a, *n* = 2,843). As a test of accuracy for the quick ages, a subset of these zircons, mostly Hadean, were selected for the full U–Pb analysis. Of the 125 zircons that are over 95% concordant, the U–Pb ages are on average only 4 Myr different from quick ages, including some grains with large age differences that may be zoned (Extended Data Fig. 1b).

As in other studies of JH zircons^1,2,75^, most of the zircons from 01JH-12 are Archean. The dominant population has an average age of 3.4 Gyr (Extended Data Fig. 1a), similar to gneisses surrounding the JH. Curiously, there are few zircons dated to between 3.7 Ga, the age of the oldest known rocks in Australia, and 3.9 Ga. The Hadean zircons represent a different subset with a peak at 4 Ga extending to 4.4 Ga for concordant U–Pb ages. Most of the Hadean ages were verified by a full U–Pb analysis. Of the 2,843 quick ages in mounts ERC-8, ERC-9 and ERC-10, there are 300 older than 3.9 Gyr and 218 older than 4.0 Gyr (10.6% and 7.8%, respectively). Moreover, a small number of zircons with quick ages <4.0 Gyr are shown by full U–Pb analyses to be older than 4.0 Gyr, indicating that the actual percentage of Hadean zircons in the sample is 8.8%.

The reported percentages of Hadean (>4.0 Ga) ages in other studies of the JH zircons range from about 3 to 12% among concordant zircons^1,2,12,25–27,46,75,76^. Even at 3%, this is a notably higher percentage than at any other known locality^77,78^. Ages younger than 3.0 Gyr, the putative age of sedimentation, have been reported from JH metasediments and could result from contamination in the field^79^ or otherwise unrecognized differences in lithology^25^. These young ages were not encountered in this study (with one exception) and are ignored in other databases.

### Geodynamic modelling

**Extended Data Fig. 2:**
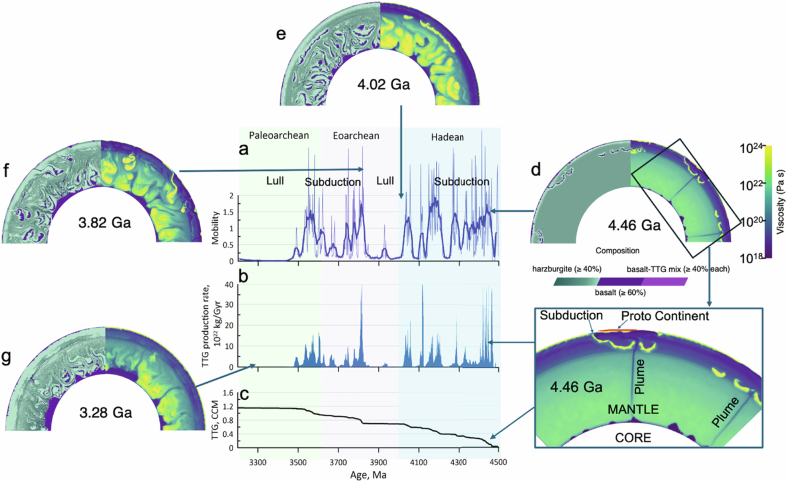
Geodynamic model. Geodynamic model of early Earth evolution^37^. **2a**. Mobility, i.e., the ratio of root mean square (RMS) of surface velocity and RMS of velocity of the entire mantle over time with a moving average (thick line) over 11 mobility values (thin lines). A value of mobility above one indicates subduction activity. **2b,c**. The rate and mass of granitic continental crust (TTG) generated over time, respectively, the latter scaled with the present-day continental crust mass (CCM). **2d,e,f** and **g**. Snapshots of Earth’s cross-section showing the modeled evolution with time, where the two quadrants represent different fields (left: composition, right: viscosity). The color scales for each field are shown in **d**. Figures **d** and **f** show periods of active subduction, while Figures **e** and **g** show lulls in subduction activity where large mantle plumes (dark blue in the viscosity field) are trapped beneath cold recycled material (yellow in the viscosity field). The magnified inset from **d** shows subduction beneath proto-continental crust in the Early Hadean.

**Extended Data Fig. 3:**
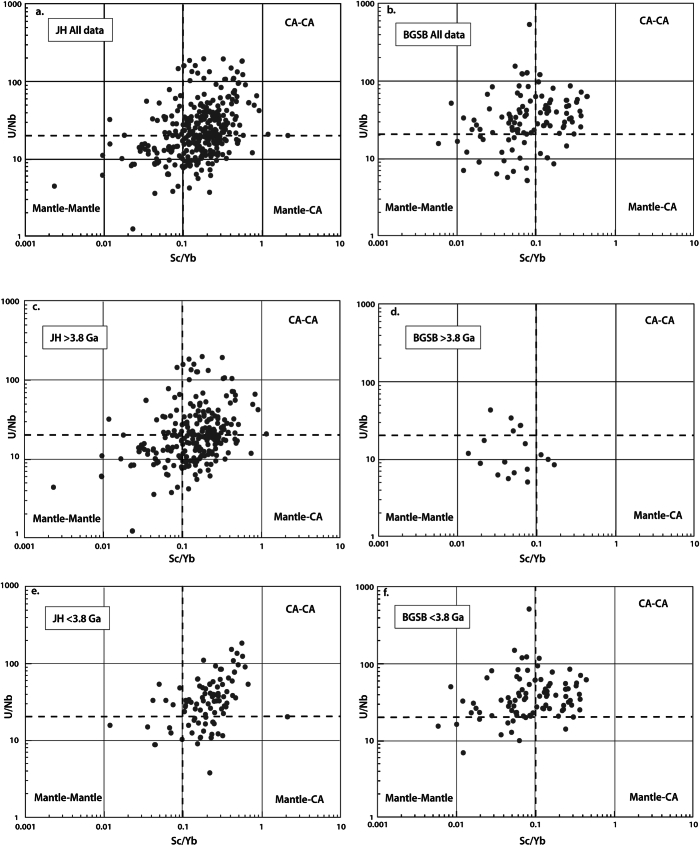
Sc/Yb vs. U/Nb for zircons. Plots of Sc/Yb vs. U/Nb for detrital zircons from Jack Hills metasediments (this study) and the Barberton Green Sandstone Bed^13^. a&b show all data, c&d older than 3.8 Ga, and e&f younger than 3.8 Ga. Discriminants for Continental Arcs/ Subduction (CAS) vs. Mantle from Grimes et al.^14^. Note that a significant proportion of Hadean zircons have mantle-like U/Nb and CAS-like Sc/Yb (lower-right quadrant) due to hydrous melting conditions and different mineralogy, see text. Such mantle-CAS zircons are less common after 3.8 Ga.

Previous studies^37,44^ applied the mantle convection code StagYY^80^ to model the Hadean and Eoarchean Earth in a two-dimensional spherical annulus geometry^81^ (Extended Data Fig. 2). These models incorporate pressure- and temperature-dependent water solubility maps for different mantle minerals^82^, composite rheology (diffusion creep and dislocation creep proxy), the effect of water on the density of mantle minerals and the frictional strength of the oceanic lithosphere of early Earth to estimate the production of tonalite, trondhjemite and granodiorite magmas and the evolution of trace elements including U and Nb.

## Online content

Any methods, additional references, Nature Portfolio reporting summaries, source data, extended data, supplementary information, acknowledgements, peer review information; details of author contributions and competing interests; and statements of data and code availability are available at 10.1038/s41586-025-10066-2.

## Supplementary information

Supplementary Table 1 Age, oxygen isotope and trace element data for JH zircons from samples 01JH-12 (mounts ERC-2 to ERC-10). Trace elements are also reported for zircons from 01JH-13(a,b), 01JH-36, 01JH-42, 01JH-47, 01JH-54(a,b), 01JH-60(a,b) and W-74-3 and W-74-4. Other data are summarized in ref. ^26^. Available from EarthChem: https://doi.org/10.60520/IEDA/114181.

Supplementary Table 2 Trace element standards run in three sessions in 2022–2024

## Data Availability

Age and δ^18^O for samples in mounts prefixed by 01JH or W were reported earlier^26^. ERC mounts were analysed for age, δ^18^O and trace elements in this study (Supplementary Table 1). Tables of age, oxygen isotope ratios and trace elements in JH zircons of this study are available at EarthChem: 10.60520/IEDA/114181.
